# Self-efficacy and oral health-related quality of life among immigrants in Southeast Iran: a cross-sectional study

**DOI:** 10.1186/s12903-025-07508-8

**Published:** 2025-12-12

**Authors:** Hossein Izadirad, Maryam Rastegar, Saideh Koshafar

**Affiliations:** 1 Health Promotion Research Center, Zahedan University of Medical Sciences, Zahedan, Iran; 2 Department of Epidemiology and Biostatistics, School of Health, Zahedan University of Medical Sciences, Zahedan, Iran; 3 Infectious Diseases and Tropical Medicine Research Center, Research Institute of Cellular and Molecular Sciences in Infectious Diseases, Zahedan University of Medical Sciences, Zahedan, Iran; 4 BSc. Student Research Center, Zahedan University of Medical Sciences, Zahedan, Iran

**Keywords:** Oral health self-efficacy, Oral health, Quality of life, Immigrants

## Abstract

**Background:**

Oral health is a key component of overall well-being. This study examined the relationship between self-efficacy and oral health-related quality of life (OHRQoL) among Afghan immigrants in Southeast Iran.

**Methods:**

In this cross-sectional study, 400 immigrants were surveyed using convenience sampling. Data were collected using validated questionnaires on oral health self-efficacy (OHSQ) and OHRQoL (OHIP-14). Data analysis included Pearson correlation, one-way ANOVA, independent t-tests and multiple linear regression.

**Results:**

The mean age of participants was 31.22 ± 14.03 years. A significant disparity was observed in self-efficacy scores, with the overall mean at 2.81 (SD = 3.09), brushing at 2.76 (SD = 3.02), and flossing notably low at 0.05 (SD = 0.40). The mean OHIP-14 score of 19.46 ± 8.01 indicated a moderate level of oral health-related impairment. Higher education and income were significantly associated with greater self-efficacy, while female gender and lower educational levels were associated with poorer OHRQoL. Critically, a significant negative correlation was found between overall self-efficacy and OHIP-14 scores, supporting the association between higher self-efficacy and better OHRQoL.

**Conclusion:**

This study confirms that higher self-efficacy correlates with better oral health in Afghan immigrants in Iran. Essential interventions include culturally-adapted education in native languages and addressing key barriers like lack of insurance and low education. Despite the cross-sectional design limitations, integrated public health strategies combining self-efficacy building and healthcare access are strongly recommended.

## Background

Oral health is a fundamental component of overall health and quality of life, with oral diseases posing a significant global public health challenge. According to the Global Burden of Disease Study 2017, untreated dental conditions affect nearly 3.5 billion people worldwide, making oral diseases the most prevalent non-communicable diseases (NCDs) and contributing substantially to years lived with disability (YLDs) [1, 2]. Oral diseases are among the most common diseases worldwide, imposing a significant health and economic burden and substantially impairing quality of life. According to the comprehensive Global Burden of Disease Study (GBD 2019), the direct treatment costs and indirect productivity losses due to oral diseases amount to hundreds of billions of US dollars annually, placing a heavy economic strain on healthcare systems and societies [3].

The burden of oral diseases is not distributed equitably. Significant disparities exist, with vulnerable populations, including migrants, facing disproportionate challenges. Migrants often encounter substantial barriers in accessing quality oral healthcare, including financial constraints, cultural and language differences, and lack of health insurance, which collectively contribute to poorer oral health outcomes compared to host populations [4, 5]. These burdens are disproportionately borne by low-income minority groups, including refugees and immigrants, who are at the highest risk [6]. The process of acculturation adds a layer of complexity to the oral health of migrant populations. Evidence indicates that lower levels of acculturation are frequently associated with worse oral health status, potentially due to increased stress, social isolation, and unfamiliarity with the host country’s healthcare system [7, 8]. Studies have shown that lower levels of acculturation are associated with higher rates of dental caries [9]. Furthermore, the age at migration affects dental caries status: older immigrants tend to have worse dental health outcomes [9–11].

Beyond structural and social factors, psychological determinants are crucial in understanding oral health behaviors. According to Social Cognitive Theory, self-efficacy—an individual’s belief in their capability to organize and execute courses of action required to achieve specific goals—is a cornerstone of health behavior change [4]. Self-efficacy is a critical psychological determinant that directly influences health behaviors and, consequently, health-related quality of life. Higher self-efficacy empowers individuals to adopt and maintain healthy behaviors, overcome challenges, and cope with health-related stressors, thereby leading to improved functional status and well-being [12, 13]. Contemporary evidence from numerous studies confirms that higher self-efficacy, both general and task-specific (e.g., toothbrushing self-efficacy), is directly associated with the adoption and maintenance of preventive oral health behaviors and better oral health-related quality of life (OHRQoL) [8, 14].

The relationship between self-efficacy and oral health is not a simple one-way street but rather a dynamic bidirectional interplay. On the one hand, higher self-efficacy can lead to improved oral health outcomes by facilitating healthy behaviors [15]. On the other hand, experiencing oral health problems can foster feelings of helplessness and undermine an individual’s self-efficacy [16, 17]. This dynamic is profoundly influenced by the process of acculturation in migrant populations. Migration-related stresses, social isolation, and language barriers can deplete the psychological resources necessary to maintain self-efficacy, consequently impacting oral health [4, 11].

The study focuses on Afghan immigrants in Southeast Iran, one of the largest and most marginalized migrant populations in the region, characterized by distinct cultural and linguistic barriers, severe economic constraints, and near-total exclusion from formal health insurance systems [18]. These specific socioeconomic and access-related vulnerabilities underscore the critical need to investigate oral health self-efficacy and OHRQoL in this underserved community.

Despite robust evidence on the roles of self-efficacy and acculturation in Western contexts, a significant research gap exists regarding the Afghan migrant population in Iran. Specifically, little is known about the relationship between self-efficacy and Oral Health-Related Quality of Life (OHRQoL) in this unique cultural and healthcare setting. Most existing research has been conducted within the socio-cultural context of Western countries, and the generalizability of their findings to the Iranian context, with its unique cultural characteristics and healthcare system structure, remains uncertain. Therefore, this study was designed to address this gap by examining the relationship between self-efficacy and OHRQoL among Afghan immigrants in Southeast Iran.

This study specifically aims to examine the relationship between self-efficacy and OHRQoL in these migrants. We hypothesize that higher levels of self-efficacy will be significantly associated with better OHRQoL scores. The findings of this study can provide the scientific foundation for developing culturally-sensitive, self-efficacy-enhancing interventions specifically tailored to improve the oral health of this vulnerable population.

## Methods

### Study design and population

This cross-sectional study was conducted from March to June 2023 among Afghan immigrants residing in southeastern Iran. The study population consisted of adult immigrants (aged 18–65 years) living in cities covered by Zahedan University of Medical Sciences, including the cities of Zahedan, Khash, Saravan, Mirjaveh, and Sib-Suran.

### Sample size calculation

Based on a similar previous study [19] and using the formula below with Z = 1.96 (for a 95% confidence level), *P* = 0.63, and d = 0.05, the sample size was calculated as 358 participants. A final sample of 400 was used to account for potential non-response.

$$\:n=\frac{{Z}^{2}.P.(1-P)}{{d}^{2}}=358$$

The samples were selected using a non-probability convenience sampling method. Specifically, March to June 2023 among Afghan immigrants residing in southeastern Iran, the required sample was selected from eligible immigrant visitors at three urban community health service centers. After obtaining written informed consent, the researcher proceeded to complete the questionnaire and collect data. Inclusion criteria were: age 18 to 65 years, immigrant status, a minimum of one year of residence in the study area. Exclusion criteria were having psychological problems and disorders, and having dentures. All participants completed a written consent form before the study began, and they were fully assured of the confidentiality of their responses.

### Sampling method

Participants were selected through convenience sampling from eligible visitors to urban comprehensive health service centers. In each city, one comprehensive health service center was randomly selected, and from each selected center, 80 eligible migrants were recruited. We acknowledge that this non-probability sampling method may introduce selection bias and limit the generalizability of our findings.

### Data collection tools

The data collection tools included the *Oral Health Impact Profile-14 (OHIP-14)* and the *Oral Health Self-Efficacy Questionnaire (OHSQ)*.

#### OHIP-14

The short-form Persian version of the Oral Health Impact Profile (OHIP-14) was used to assess the oral health-related quality of life of the participating immigrants. This questionnaire has demonstrated acceptable validity and reliability and has been utilized in numerous studies in Iran. It consists of 7 domains: functional limitation, physical pain, psychological discomfort, physical disability, psychological disability, social disability, and overall disability. Each item in the instrument is scored on a scale of 0 to 4. A score of 0 indicates good quality of life, while a score of 4 indicates the worst condition. Thus, the total score ranges from 0 to 56 [20].

In the original validation study by Slade (1997), the OHIP-14 demonstrated acceptable reliability and validity. The internal consistency (Cronbach’s alpha) was 0.88, and the test-retest reliability (intraclass correlation coefficient) over a two-week interval was 0.82. Construct validity was established through significant correlations with clinical oral health indicators (including the number of missing teeth and self-rated oral health). The instrument, structured across seven domains, demonstrated the ability to discriminate between groups with different oral health statuses [20].

The referenced study demonstrated that the Persian version of the OHIP-14 questionnaire has acceptable reliability and validity. Cronbach’s alpha coefficient was 0.954 for the entire questionnaire and ranged from 0.770 to 0.871 for the seven domains. Furthermore, criterion validity was confirmed through significant correlations between OHIP scores and dental treatment needs (*p* < 0.0001) [21].

 The Oral Health Self-Efficacy Questionnaire (OHSQ) was developed based on Bandura’s theoretical framework of self-efficacy to measure individuals’ confidence [22] to perform a specific task. The questionnaire contains 14 items divided into two subscales: toothbrushing self-efficacy (7 items) and flossing self-efficacy (7 items). Items assess confidence in maintaining oral hygiene under challenging circumstances (e.g., when feeling tired, busy, or unmotivated).

Scoring: All items are scored on a Likert scale from 0 (not confident at all) to 1 (confident). Subscale scores are calculated by summing the respective items (potential range: 0–7 for each subscale), with higher scores indicating greater self-efficacy. The total score ranges from 0 to 14, representing overall oral health self-efficacy.

Psychometric Properties: In the development study, the instrument demonstrated acceptable reliability. The toothbrushing subscale showed good internal consistency (item-total correlations: 0.78–0.86; mean = 0.82) as did the flossing subscale (item-total correlations: 0.62–0.85; mean = 0.76). Test-retest reliability over a two-week interval was moderate (toothbrushing: *r* = 0.62; flossing: *r* = 0.60) [22].

### Data analysis

All statistical analyses were conducted using SPSS version 26 (IBM Corp., Armonk, NY, USA). Descriptive statistics, including means, standard deviations, frequencies, and percentages, were used to summarize demographic characteristics, self-efficacy scores, and oral health–related quality of life.

The oral health self-efficacy questionnaire consisted of 14 dichotomous items, including seven items related to toothbrushing and seven items related to flossing. Each item was scored as 1 = “Yes” (confident to perform the behavior) and 0 = “No” (not confident). Brushing and flossing self-efficacy scores were calculated by summing the respective items, yielding a range of 0 to 7 for each subscale. The overall self-efficacy score was obtained by summing all 14 items, with a possible range of 0 to 14, where higher scores indicated greater perceived self-efficacy in performing oral hygiene behaviors.

The OHIP-14 questionnaire was scored according to the standard method, with response options from 0 (“never”) to 4 (“very often”). The total OHIP score ranged from 0 to 56, with higher scores reflecting poorer oral health–related quality of life. Subscale scores for the seven OHIP domains were also computed.

Categorical variables were coded before analysis as follows: sex (0 = male, 1 = female), marital status (0 = single, 1 = married), education level (0 = illiterate, 1 = primary/secondary, 2 = high school/diploma or higher), occupation (unemployed, housekeeper, self-employed, worker), and health insurance (0 = no, 1 = yes). Continuous variables (age, monthly income, number of children, and years of residence) were treated as numerical predictors.

Group differences in self-efficacy and OHIP scores were assessed using independent t-tests for two-category variables and one-way ANOVA for variables with more than two categories. When ANOVA results were statistically significant, Tukey’s post-hoc test was used to identify pairwise differences. Assumptions of normality (Shapiro–Wilk) and homogeneity of variances (Levene’s test) were checked prior to conducting parametric tests.

Pearson correlation coefficients were used to examine associations among continuous variables such as age, income, self-efficacy scores, and OHIP outcomes. To evaluate the predictive role of self-efficacy in oral health–related quality of life, a multiple linear regression model was constructed with the overall OHIP score as the dependent variable. Independent variables included overall self-efficacy score, age, sex, education level, and income. A forced-entry method was applied, and multicollinearity was assessed using variance inflation factors (VIF), all of which were within acceptable ranges.

No missing data were present, as questionnaires were checked for completeness during data collection. A p-value of less than 0.05 was considered statistically significant for all tests.

#### Ethical considerations

The study was approved by the Ethics Committee of Zahedan University of Medical Sciences (code: IR.ZAUMS.REC.1402.136). Written informed consent was obtained from all participants.

## Result

The results indicate that the immigrant population in Southeast Iran has a mean age of 31.22 years (SD = 14.03), suggesting a relatively young group with considerable age variation. The study investigating self-efficacy and oral health outcomes among the immigrant population of Southeast Iran in 2023 provides significant demographic insights. The findings indicate that the majority of participants were women (59.5%). Additionally, most individuals were married (83.3%), suggesting a predominantly family-oriented community. Educational attainment was notably low, as 65.3% of the participants were illiterate.

Employment data revealed that the largest occupational category was housekeepers (36.5%), followed by self-employed individuals (34.5%) and workers (15.3%). A notable proportion of the population was unemployed (13.5%). A notable finding of the study was that none of the participants reported having health insurance (0%), indicating a complete lack of formal health coverage within the sample. The average time of residence is 24 years (SD = 13), reflecting a mix of long-term and recent immigrants. Additionally, the mean number of children is 3.43 (SD = 3.45), showing significant variability in family size. (Table 1).

**Table 1:** Demographic characteristics of the immigrant population in Southeast Iran (*n* = 400)

variable	category	*N*	*N* %
Sex	Men	162	40.5%
	Women	238	59.5%
Marital status	Single	67	16.8%
	Married	333	83.3%
Education	Illiterate	261	65.3%
	High school	136	34.0%
	Diploma and Graduate	3	1.0%
Religion	Shia	0	0.0%
	Sunni	400	100.0%
Job	Workless	54	13.5%
	Housekeeper	146	36.5%
	Self-employment	138	34.5%
	Business	1	0.3%
	Worker	61	15.3%
	Employee	0	0.0%
Insurance	Yes	0	0.0%
	No	400	100.0%
Age (year)	Mean ± SD^*^	31.22 ± 14.03	
time of residence (year)	Mean ± SD	24 ± 13	
Income (million Toman)	Mean ± SD	8.66 ± 3.76	
Number of children	Mean ± SD	3.43 ± 3.45	

*SD: Standard Deviation

Table 2 presents the self-efficacy and oral health-related quality of life (OHIP) domain scores among the immigrant population of Southeast Iran in 2023. The mean self-efficacy score was 2.81 (SD = 3.09), with brushing self-efficacy averaging 2.76 (SD = 3.02) and flossing self-efficacy being notably low at 0.05 (SD = 0.40). This suggests a significant disparity in confidence between brushing and flossing behaviors, indicating a potential gap in oral hygiene practices.

**Table 2:** Self-Efficacy and OHIP domain scores in the immigrant population of Southeast Iran (2023)

Domain	Mean ± SD*	Minimum	Maximum
Self-Efficacy (Brushing)	2.76 ± 3.02	0.00	7.00
Self-Efficacy (Flossing)	0.05 ± 0.40	0.00	7.00
Overall Self-Efficacy	2.81 ± 3.09	0.00	14.00
Functional Limitation	2.16 ± 0.70	2.00	8.00
Physical Pain	4.93 ± 2.76	2.00	10.00
Psychological Discomfort	0.01 ± 0.10	0.00	2.00
Physical Disability	2.88 ± 1.74	2.00	10.00
Psychological Disability	3.07 ± 1.33	2.00	9.00
Social Disability	3.19 ± 1.60	2.00	10.00
Handicap	3.23 ± 1.60	2.00	10.00
Overall OHIP Score	19.46 ± 8.01	12.00	49.00

* SD: Standard Deviation, OHIP: Oral Health Impact Profile

The overall OHIP score was 19.46 (SD = 8.01), reflecting the frequency of oral health-related issues across different domains. The highest mean score was observed in physical pain (4.93 ± 2.76), followed by handicap (3.23 ± 1.60), social disability (3.19 ± 1.60), and psychological disability (3.07 ± 1.33), suggesting that pain and functional limitations are prominent concerns. Psychological discomfort had the lowest mean score (0.01 ± 0.10), indicating minimal reported distress in this domain.

Table 3 presents the mean self-efficacy scores, including brushing and flossing self-efficacy, in the immigrant population of Southeast Iran in 2023, categorized by demographic variables such as sex, marital status, education, and job status. The results indicate that there is no statistically significant difference in self-efficacy scores between men and women across all domains. Similarly, marital status does not appear to have a significant association on self-efficacy, although single individuals exhibit slightly higher mean scores compared to married individuals.

**Table 3:** Mean Self-Efficacy score in the immigrant population of Southeast Iran (2023) categorized by demographic variables

Variable	Self-Efficacy (Brushing)		Self-Efficacy (Flossing)		Self-Efficacy	
Sex	Mean ± SD	p-value	Mean ± SD	p-value	Mean ± SD	p-value
Men	2.83 ± 3.06	0.325^*^	0.02 ± 0.14	0.529^*^	2.85 ± 3.09	0.461^*^
Women	2.71 ± 2.99		0.07 ± 0.50		2.79 ± 3.09	
Marital Status						
Single	3.01 ± 3.07	0.074^*^	0.03 ± 0.17	0.936^*^	3.04 ± 3.11	0.396^*^
Married	2.71 ± 3.01		0.05 ± 0.43		2.77 ± 3.09	
Education						
Illiterate	2.48 ± 2.93	0.607^**^	0.03 ± 0.18	0.016^**^	2.50 ± 2.96	0.048^**^
High School	3.22 ± 3.09		0.09 ± 0.63		3.31 ± 3.23	
Diploma	7.00 ± 0.00		0.50 ± 0.71		7.50 ± 0.71	
Graduate	7.00 ± 0.00		0.03 ± 0.10		7.00 ± 0.00	
Job						
Workless	2.74 ± 3.09	0.079^**^	0.02 ± 0.14	0.75^**^	2.76 ± 3.12	0.203^**^
Housekeeper	2.40 ± 2.87		0.08 ± 0.62		2.49 ± 3.01	
Self-Employment	3.51 ± 3.17		0.05 ± 0.22		3.57 ± 3.22	
Business	7.00 ± 0.00		0.00 ± 0.00		7.00 ± 0.00	
Worker	1.87 ± 2.55		0.00 ± 0.00		1.87 ± 2.55	
	correlation	p-value^***^	correlation	p-value^***^	correlation	p-value^***^
Age	−0.075	0.134	−0.045	0.373	−0.079	0.115
time of residence	−0.070	0.161	−0.034	0.500	−0.073	0.145
Income	0.104	0.038	0.072	0.153	0.111*	0.027
Number of children	−0.038	0.446	−0.036	0.477	−0.042	0.403

*Independent T-test, ** one-way ANOVA, *** Pearson correlation

Education level, however, is a significant determinant of self-efficacy, with individuals possessing a diploma or higher demonstrating markedly higher scores compared to those with lower education levels. The statistical significance observed in overall self-efficacy (*p* = 0.048) and flossing self-efficacy (*p* = 0.016) suggests that educational attainment positively influences self-efficacy in oral health behaviors. Regarding occupational status, individuals engaged in self-employment or business report the highest self-efficacy scores, whereas housekeepers and workers display lower levels. However, these differences are not statistically significant. Positive correlations were found between income and brushing self-efficacy (*p* = 0.038) and overall self-efficacy (*p* = 0.027). The correlation with age, time of residence, and number of children was not significant for any of the variables.

The study reveals significant demographic variations in health-related limitations. Women reported higher levels of physical pain (*p* = 0.013), psychological disability (*p* = 0.001), and social disability (*p* = 0.058), compared to men. Married individuals exhibited higher levels of functional limitations (*p* = 0.047), physical pain (*p* = 0.009), physical disability (*p* = 0.015), psychological disability (*p* = 0.007), social disability (*p* = 0.039), and handicap (*p* = 0.021) than singles, except for psychological discomfort (*p* = 0.654). Education level was strongly associated with limitations, with illiterate individuals reporting greater distress in all categories (p-values ranged from 0.003 to 0.000). Occupation influenced psychological disability (*p* = 0.002), with unemployed or self-employed individuals showing higher levels of psychological distress. These findings underscore the impact of demographic factors on health outcomes, particularly marital status, gender, education, and occupation (Table 4).

**Table 4:** Association between demographic characteristics and Health-Related limitations in a study population

Variable	Functional Limitation	Physical Pain	Psychological Discomfort	Physical Disability	Psychological Disability	Social Disability	Handicap
Sex							
Man	2.17 ± 0.84	4.51 ± 2.72	0.00 ± 0.00	2.73 ± 1.63	2.80 ± 1.10	3.01 ± 1.62	3.10 ± 1.66
Woman	2.15 ± 0.59	5.21 ± 2.76	0.01 ± 0.13	2.97 ± 1.81	3.26 ± 1.43	3.32 ± 1.58	3.32 ± 1.55
*p*-value^*^	0.784	0.013	0.410	0.184	0.001	0.058	0.188
Marital Status							
Single	2.00 ± 0.00	4.12 ± 2.55	0.00 ± 0.00	2.40 ± 1.09	2.67 ± 1.02	2.82 ± 1.41	2.82 ± 1.45
Married	2.19 ± 0.77	5.09 ± 2.77	0.01 ± 0.11	2.97 ± 1.83	3.15 ± 1.37	3.26 ± 1.63	3.32 ± 1.61
*p*-value^*^	0.047	0.009	0.654	0.015	0.007	0.039	0.021
Education							
Illiterate	2.21 ± 0.84	5.29 ± 2.80	0.00 ± 0.00	3.12 ± 1.94	3.28 ± 1.45	3.38 ± 1.67	3.41 ± 1.67
High School	2.04 ± 0.27	4.26 ± 2.57	0.01 ± 0.17	2.42 ± 1.19	2.68 ± 0.93	2.86 ± 1.42	2.91 ± 1.41
Diploma	2.00 ± 0.00	3.00 ± 1.41	0.00 ± 0.00	2.00 ± 0.00	2.50 ± 0.71	2.00 ± 0.00	2.00 ± 0.00
Graduate	2.00 ± 0.00	3.00 ± 0.01	0.00 ± 0.00	2.00 ± 0.01	2.00 ± 0.02	2.00 ± 0.01	2.00 ± 0.01
*p*-value^**^	0.143	0.003	0.586	0.001	0.000	0.012	0.013
Job							
Workless	2.41 ± 1.32	5.06 ± 2.67	0.00 ± 0.00	3.33 ± 2.23	3.33 ± 1.61	3.54 ± 1.72	3.52 ± 1.76
Housekeeper	2.16 ± 0.48	5.25 ± 2.78	0.01 ± 0.17	2.94 ± 1.80	3.34 ± 1.46	3.38 ± 1.65	3.36 ± 1.57
Self-Employment	2.04 ± 0.32	4.76 ± 2.79	0.00 ± 0.00	2.72 ± 1.53	2.83 ± 1.04	2.97 ± 1.43	3.08 ± 1.49
Business	2.00 ± 0.01	2.00 ± 0.01	0.00 ± 0.00	2.00 ± 0.00	2.00 ± 0.00	2.00 ± 0.00	2.00 ± 0.00
Worker	2.16 ± 0.92	4.44 ± 2.69	0.00 ± 0.00	2.67 ± 1.54	2.77 ± 1.13	2.95 ± 1.69	3.03 ± 1.74
*p*-value^**^	0.031	0.226	0.785	0.197	0.002	0.060	0.238

*Independent T-test, ** one way ANOVA

Table 5 presents the Pearson correlation coefficients examining the relationship between self-efficacy (in brushing, flossing, and overall) and various domains of oral health-related quality of life (OHRQoL). The findings indicate that brushing self-efficacy was significantly and negatively associated with several OHRQoL domains, including functional limitation (*r* = −0.167, *p* = 0.001), physical pain (*r* = −0.189, *p* < 0.001), physical disability (*r* = −0.148, *p* = 0.003), psychological disability (*r* = −0.215, *p* < 0.001), social disability (*r* = −0.175, *p* < 0.001), and handicap (*r* = −0.164, *p* = 0.001). These negative correlations suggest that individuals with higher confidence in their toothbrushing ability tend to experience fewer oral health-related impairments and disabilities. Similarly, overall self-efficacy, which reflects combined confidence in both brushing and flossing, showed a statistically significant negative correlation with all the same domains, supporting the notion that higher self-efficacy is linked to better perceived oral health outcomes. In contrast, flossing self-efficacy did not show statistically significant correlations with any of the OHRQoL domains. Although a weak positive correlation was observed between flossing self-efficacy and psychological discomfort (*r* = 0.087, *p* = 0.068), it did not reach statistical significance.

**Table 5:** Relationship between Self-Efficacy and oral health outcomes in the immigrant population of Southeast Iran (2023)

Variable	Self-Efficacy (Brushing)		Self-Efficacy (Flossing)		Self-Efficacy	
	Correlation^*^	*p*-value	Correlation	*p*-value	Correlation	*p*-value
Functional Limitation	−0.167	0.001	−0.001	0.986	−0.164	0.001
Physical Pain	− 0.189	*p* < 0.001	−0.061	0.227	− 0.193	*p* < 0.001
Psychological Discomfort	0.070	0.160	0.087	0.068	0.081	0.071
Physical Disability	− 0.148	0.003	−0.034	0.493	− 0.149	0.003
Psychological Disability	− 0.215	*p* < 0.001	−0.064	0.201	− 0.218	*p* < 0.001
Social Disability	− 0.175	*p* < 0.001	−0.070	0.162	− 0.180	*p* < 0.001
Handicap	− 0.164	0.001	−0.074	0.141	− 0.170	0.001

* Pearson correlation

The regression model significantly predicted brushing self-efficacy, although the explained variance was small (Adjusted R² = 0.048). Among the OHIP domains, Functional Limitation was the only significant negative predictor (*p* = 0.020), indicating that greater functional problems were associated with lower brushing confidence. Other domains were not significant predictors (Table 6). The model explained 77% of the variance in flossing self-efficacy, indicating a very strong predictive relationship. The only significant predictor was Psychological Discomfort, which showed a very strong positive association (β = 0.876, *p* < 0.001). This suggests that individuals reporting higher psychological discomfort unexpectedly showed higher flossing self-efficacy — possibly reflecting measurement bias or the extremely skewed distribution of flossing scores (mostly zeros) (Table 7). The multiple regression model significantly predicted overall oral health self-efficacy among immigrants; however, it accounted for a relatively small proportion of the variance in self-efficacy scores (Adjusted R² = 0.076). Among the OHIP domains entered into the model, Functional Limitation emerged as a significant negative predictor of self-efficacy (*p* = 0.023), indicating that individuals experiencing greater functional problems reported lower confidence in performing oral hygiene behaviors. In contrast, Psychological Discomfort showed a significant positive association with overall self-efficacy (*p* < 0.001). None of the remaining OHIP domains—including physical pain, physical disability, psychological disability, social disability, or handicap—demonstrated a statistically significant contribution to the prediction of self-efficacy. These findings suggest that while certain aspects of oral health–related quality of life are related to perceived self-efficacy, the overall predictive strength of OHIP domains remains modest (Table 8).

**Table 6:** Multiple linear regression predicting brushing Self-Efficacy in the immigrant population of Southeast Iran (2023)

Predictor	B	SE	β	t	*p*-value
Constant	5.193	0.557	—	9.321	< 0.001
Functional Limitation	–0.563	0.241	–0.131	–2.333	0.020
Physical Pain	–0.084	0.105	–0.077	–0.802	0.423
Psychological Discomfort	1.893	1.476	0.063	1.283	0.200
Physical Disability	0.039	0.113	0.022	0.343	0.732
Psychological Disability	–0.247	0.205	–0.109	–1.208	0.228
Social Disability	0.054	0.3	0.029	0.18	0.857
Handicap	–0.104	0.289	–0.055	–0.360	0.719

Model fit: *R* = 0.255, *R²* = 0.065, Adjusted *R²* = 0.048, F(7,392) = 3.89

*p* < 0.001

**Table 7:** Multiple linear regression predicting flossing Self-Efficacy in the immigrant population of Southeast Iran (2023)

Predictor	B	SE	β	t	*p*-value
Constant	0.051	0.036	—	1.413	0.159
Functional Limitation	0.005	0.016	0.009	0.334	0.739
Physical Pain	–0.007	0.007	–0.050	–1.043	0.297
Psychological Discomfort	3.481	0.096	0.876	36.193	< 0.001
Physical Disability	0.002	0.007	0.01	0.298	0.766
Psychological Disability	0.003	0.013	0.011	0.255	0.799
Social Disability	0	0.02	0	–0.002	0.999
Handicap	–0.004	0.019	–0.014	–0.189	0.851

Model fit: *R* = 0.878, *R²* = 0.771, Adjusted *R²* = 0.767, F(7,392) = 188.66

*p* < 0.001

**Table 8:** Multiple linear regression predicting overall Self-Efficacy in the immigrant population of Southeast Iran (2023)

Predictor	B	SE	β	t	*p*-value
Constant	5.244	0.562	—	9.327	< 0.001
Functional Limitation	–0.557	0.243	–0.127	–2.290	0.023
Physical Pain	–0.091	0.106	–0.082	–0.862	0.389
Psychological Discomfort	5.375	1.489	0.174	3.609	< 0.001
Physical Disability	0.041	0.114	0.023	0.359	0.72
Psychological Disability	–0.244	0.206	–0.105	–1.181	0.238
Social Disability	0.054	0.303	0.028	0.178	0.859
Handicap	–0.108	0.292	–0.056	–0.369	0.712

Model fit: *R* = 0.303, *R²* = 0.092, Adjusted *R²* = 0.076, F (7,392) = 5.66

*p* < 0.001

## Discussion

### Main findings

This study was driven by the hypothesis that higher levels of self-efficacy would be significantly associated with better Oral Health-Related Quality of Life (OHRQoL) among Afghan immigrants. Our results strongly confirm this central hypothesis. A significant negative correlation was found between overall self-efficacy and the total OHIP-14 score (*r* = −0.18, *p* < 0.001), indicating that greater confidence in one’s ability to perform oral hygiene practices is directly associated with fewer reported oral health impacts and a better quality of life. This foundational relationship underscores the critical role of psychological empowerment in the oral health of this marginalized population.

Also, the regression analysis demonstrated that oral health–related quality of life domains had a limited yet significant role in predicting oral health self-efficacy among immigrants. Although the overall model accounted for a modest proportion of the variance, two OHIP domains showed meaningful associations with self-efficacy. Functional Limitation was identified as a significant negative predictor, suggesting that individuals who experience greater difficulty in chewing, speaking, or performing basic oral functions tend to perceive themselves as less capable of maintaining oral hygiene behaviors. Conversely, Psychological Discomfort emerged as a significant positive predictor of self-efficacy. This unexpected finding may reflect underlying measurement characteristics or behavioral responses in this population, particularly given the highly skewed distribution of flossing self-efficacy scores. Other OHIP domains—including physical pain, physical and psychological disability, social disability, and handicap—were not significantly associated with self-efficacy, indicating that not all dimensions of oral health–related quality of life influence perceived behavioral capability. Overall, these results suggest that self-efficacy among immigrants may be shaped more strongly by functional challenges and psychological factors than by pain or social impacts, highlighting the need for targeted interventions that address both functional barriers and motivational components of oral health behavior.

Beyond this key finding, our study reveals that this vulnerable population experiences notably low self-efficacy, particularly regarding flossing (0.05 ± 0.40), and reports a moderate level of overall oral health impairment (OHIP-14 score: 19.46 ± 8.01). Furthermore, socioeconomic factors, including low educational attainment and income, were identified as important determinants of both self-efficacy and oral health outcomes.

### Comparison with existing literature

This study investigated self-efficacy and oral health-related quality of life (OHRQoL) among the immigrant population in Southeast Iran, revealing several important findings that align with and contribute to the existing literature. The predominance of female participants (59.5%) and a high rate of marital status (83.3%) reflect patterns commonly seen in migrant communities, where family structure and caregiving roles may disproportionately involve women [23]. The strikingly low levels of educational attainment, with 65.3% being illiterate, highlight a significant social determinant of health. Educational status is a well-documented predictor of both health literacy and oral health behaviors. The lack of formal employment among a large portion of respondents, especially among women who were mostly housekeepers, further underscores the socioeconomic vulnerabilities of this population [24]. A critical finding of this study was the complete absence of health insurance coverage among all participants, highlighting a significant structural barrier to accessing formal healthcare services, including preventive and routine oral health care. This lack of insurance likely limits the use of dental services and contributes to poorer oral health outcomes, as supported by similar findings in studies of underserved and immigrant populations. The data also revealed broader socioeconomic challenges, such as low educational attainment, high unemployment, and limited income, all of which are known social determinants of health that can negatively influence self-efficacy and oral health behaviors. These interrelated factors underscore the urgent need for targeted public health interventions that address not only oral health education but also improve access to care and economic stability within immigrant communities in Southeast Iran [25, 26].

#### Self-efficacy and oral health behaviors

The mean global self-efficacy score of 2.81 ± 3.09 reflects moderate confidence in oral hygiene behaviors, yet there’s a strikingly low level specifically for flossing (0.05 ± 0.40), signaling a critical gap in knowledge, access, or the perceived importance of flossing. International studies have demonstrated that self-efficacy plays a critical role in shaping oral hygiene behaviors, particularly flossing, which requires both technical skill and motivation [27, 28]. In the present study, self-efficacy for brushing, flossing, and overall self-efficacy showed no significant association with psychological discomfort. This lack of statistical significance may be attributed to the very low levels of flossing self-efficacy observed among participants, which likely limited the variability required to detect meaningful correlations [27, 28]. We observed a significant positive correlation between higher education levels and improved self-efficacy in flossing (*p* = 0.016), consistent with research highlighting how education bolsters health literacy and empowers individuals to adopt effective hygiene behaviors. According to Bandura’s self-efficacy theory (1997) [29], competence stems from previous behavioral mastery, and education provides foundational skills and knowledge that support these behaviors. Moreover, affluent individuals are more likely to have increased brushing and overall self-efficacy, reinforcing the link between economic resources and better health practices.

Contemporary intervention studies show that integrated, behavior-focused educational strategies—combining games, animations, and hands-on guidance—effectively increase both confidence and self-care behaviors [27]. A recent mixed-methods study conducted in California found that adults with higher self-efficacy were more likely to adhere to the American Dental Association (ADA) recommendations and identified self-efficacy as a key factor distinguishing compliant from non-compliant individuals [30]. Economic stability provides access to better oral care products, routine dental visits, and a broader understanding of the importance of preventive care. Evidence further suggests that individuals with higher income are more likely to seek dental care and participate in oral health education programs [31].

Additionally, access to health services, particularly dental care, is a strong determinant of oral health behaviors. Research indicates that individuals with health insurance are more likely to engage in preventive oral care, such as regular flossing and brushing, compared to uninsured individuals [32]. This highlights the need for policies that ensure underserved populations, especially immigrants, have access to affordable dental care. Studies have indicated that cultural attitudes toward oral hygiene play a critical role in shaping self-efficacy [7, 33]. Participants from cultures with less emphasis on dental care reported lower self-efficacy scores, which underscores the importance of cultural competence in designing health interventions. Collectively, these findings highlight that education, income, and behavior-specific training—particularly focused on flossing—are key levers for boosting oral self-efficacy. Approaches grounded in behavior change theories and targeted, interactive educational tools show promising results in enhancing both confidence and actual hygiene practices. Although a weak positive correlation was observed between flossing self-efficacy and psychological discomfort (*r* = 0.087, *p* = 0.068), it did not reach statistical significance.

#### Oral Health-Related Quality of Life (OHRQoL)

The mean overall OHIP-14 score of 19.46 ± 8.01 indicates a moderate level of oral health impairment, with physical pain being the most severely impacted domain, followed by handicap and social disability. These results highlight that dental issues extend far beyond physical symptoms, negatively affecting social participation and perceived life satisfaction. This finding echoes global evidence showing that marginalized groups, including immigrants, experience broader psychosocial consequences from oral health challenges [34].

#### Sociodemographic influences

Significant gender disparities were observed, with women reporting higher levels of physical pain, psychological distress, and social disability than men. This finding aligns with previous research indicating that older women often exhibit lower OHRQoL scores, even after controlling for factors such as income and marital status [35]. These disparities may stem from women’s dual caregiving responsibilities and a heightened awareness or expression of pain and emotional effects. Married participants scored worse across nearly all OHIP domains compared to single individuals. Rather than indicating protective effects, this likely reflects the cumulative burden of economic pressures, caregiving duties, and limited healthcare access typical in immigrant families. Educational level was strongly inversely associated with OHRQoL: illiterate participants consistently experienced greater functional impairment and distress. This mirrors broader findings that link low education to poor oral health and reduced healthcare access [36]. Employment status also played a significant role: those unemployed or self-employed had the highest psychological disability scores. Economic instability—whether from unemployment or lack of employer-provided insurance—limits access to routine dental care and exacerbates mental health burdens.

### Implications for policy and practice

These findings have important implications for designing targeted interventions. We recommend developing culturally-appropriate, Farsi/Dari/Pashto-language educational programs that specifically build competency in flossing and other under-practiced behaviors. Such interventions should incorporate hands-on demonstration and leverage principles from behavior change theory to enhance self-efficacy.

At the policy level, our results underscore the urgent need to expand access to affordable dental care for immigrant populations. This could include incorporating basic dental services into public health insurance schemes and establishing mobile dental clinics in immigrant-dense communities. Community-based approaches that train peer educators from within immigrant communities may be particularly effective for sustainable impact.

### Limitations and future research directions

This study has several limitations that should be considered when interpreting the results. First, the cross-sectional design prevents us from establishing causal relationships between self-efficacy and OHRQoL. Second, the use of convenience sampling, while practical for accessing a hard-to-reach population, limits the generalizability of our findings and may introduce selection bias. Third, all measures were based on self-report, which is subject to recall and social desirability biases.

Future research should employ longitudinal designs to track temporal relationships between self-efficacy and oral health outcomes in immigrant populations. Random sampling methods would enhance representativeness, while mixed-methods approaches could provide deeper insight into the cultural and psychological mechanisms underlying oral health behaviors. Intervention studies testing the efficacy of self-efficacy building programs specifically tailored for immigrant groups are also warranted.

## Conclusion

This study addresses a significant gap in the literature on oral health among immigrants in Iran by demonstrating that low self-efficacy is a key determinant of poor oral health outcomes among the Afghan immigrant population in southeastern Iran. Our findings confirm a significant association between higher self-efficacy and better oral health-related quality of life and highlight the crucial aggravating role of socioeconomic factors, including low education, income, and a complete lack of health insurance.

It is important to acknowledge the limitations of this study, including its cross-sectional design, which prevents causal inference. Despite these limitations, the results have clear practical implications. Future public health programs should prioritize integrated interventions that combine literacy-enhancing, culturally-sensitive education in Persian/Dari/Pashto to build self-efficacy—particularly in flossing—with concrete policies that ensure affordable access to dental care for immigrant communities in Iran.

## Data Availability

The datasets used and/or analyzed during the current study are available from the corresponding author upon reasonable request.

## Abbreviations

OHRQoL: Oral Health-Related Quality of Life
OHIP-14: Oral Health Impact Profile-14
OHSQ: Oral Health Self-Efficacy Questionnaire
